# Description Kelley-Seegmiller syndrome (partial HPRT deficiency manifesting as a gout-urolithiasis syndrome) in a patient of 15 years old

**DOI:** 10.1186/1546-0096-9-S1-P33

**Published:** 2011-09-14

**Authors:** SR Rodionovskaya, NZ Zokirov, ES Fedorov, IN Tsymbal, SO Salugina, MS Eliseev

**Affiliations:** 1 Children's Hospital №38 Federal Medical Biological Agency of Russia, Moscow, Russia; 2 Research Institute of Rheumatology, Russian Academy of Medical Sciences, Moscow, Russia

## Background

Partial deficiency of the enzyme hypoxanthine-guanine phosphoribosil transferase syndrome (Kelley-Seegmiller) is a rare genetic disorder manifesting as a gout-urolithiasis.

## Methods

We observed a boy 15 years old, with syndrome Kelley-Seegmiller.

## Results

**Figure 1:**
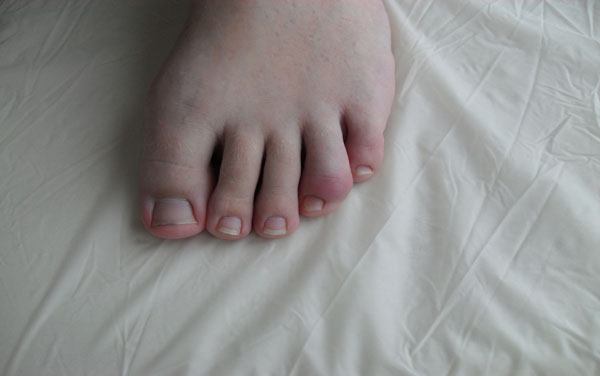


**Figure 2:**
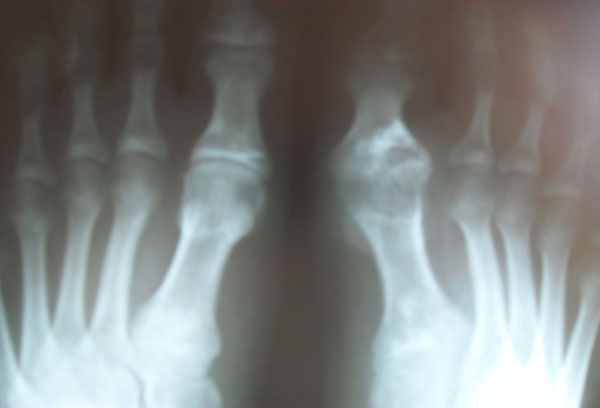


The first manifestations of the disease were identified at age of 5, there were arthralgia in the knee joints, in the heel areas. At 9 years old tophuses were found in a helix of the right ear, in the angle of the left eye. At the age of 11 there were high levels of uric acid, creatinine and urea. Severe mental disabilities are not present. After conducting genetic study a mutation in the gene Gly15Ser was revealed. The same mutation is found out in mother of the patient and the sister. Diet therapy with allopurinol, blemaren was appointed. In 15 years the patient has chronic polyarthritis including of small joints of hands and feet, knees and has spread tophuses. X-ray study has shown the destructive changes of the affected joints. Ultrasound study of kidneys has shown the symptoms of nephrolithiasis. Scintigraphy has shown the decrease in the total volume of functioning parenchyma and diffuse-focal changes of kidneys. Blood test revealed elevated levels of uric acid, creatinine, urea. Uric acid level is controlled by allopurinol therapy. Figures 1 and 2.

## Conclusions

Chronic urate tubulointerstitial nephritis and nephrocalcinosis affects the prognosis of Kelley-Seegmiller Syndrome.

